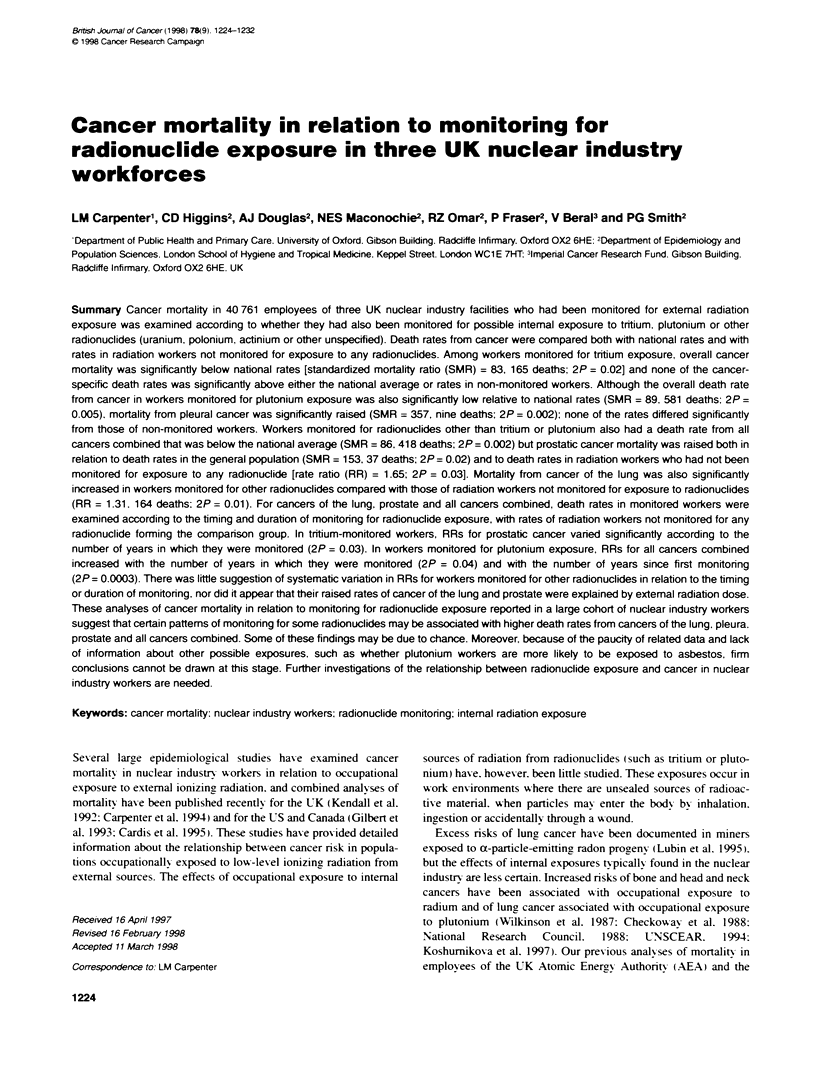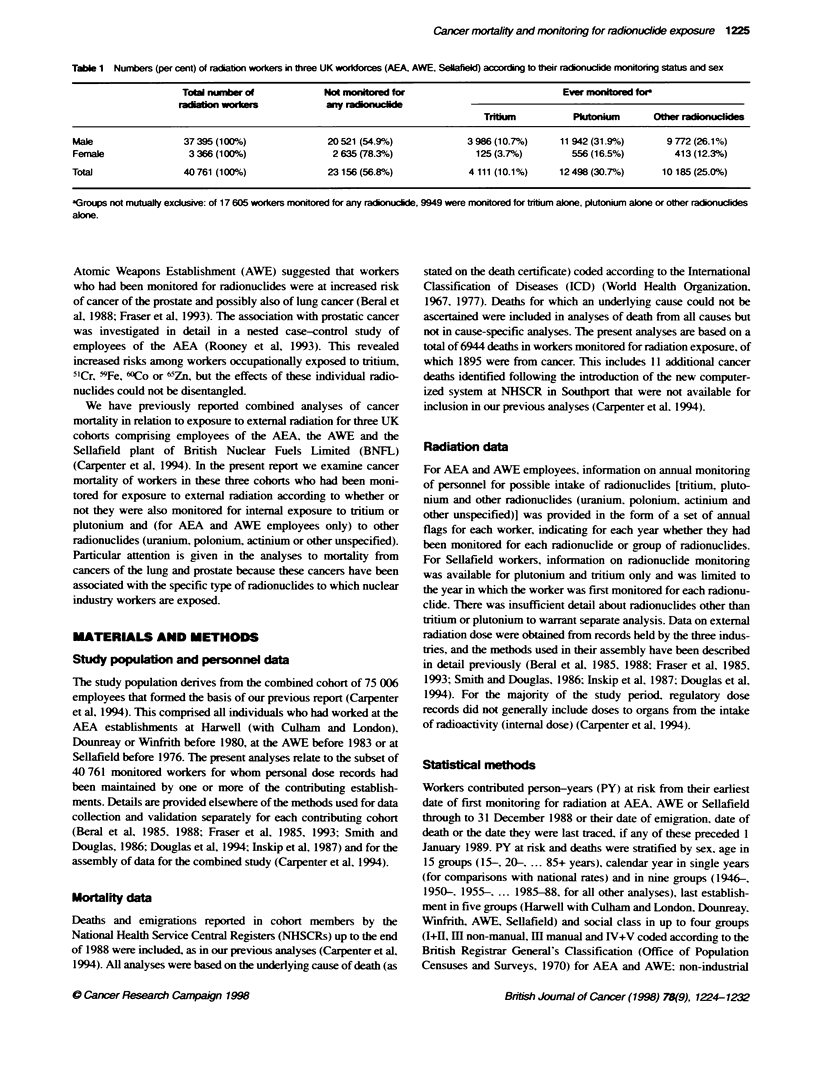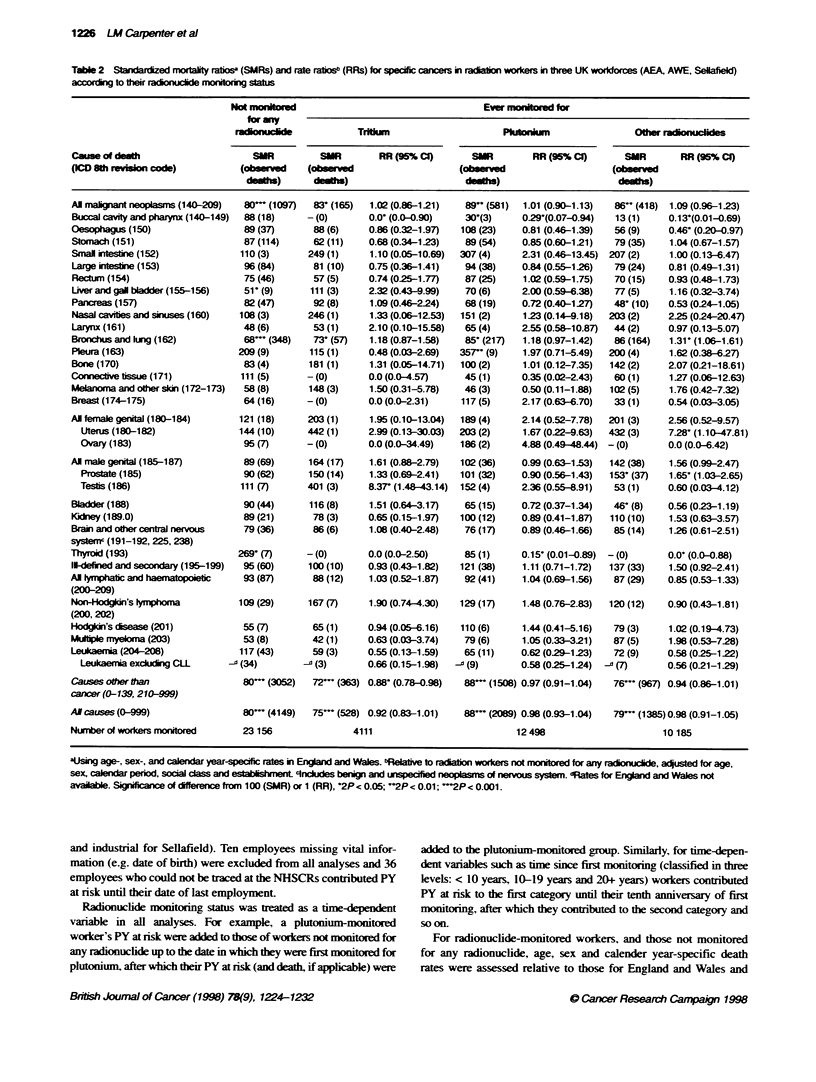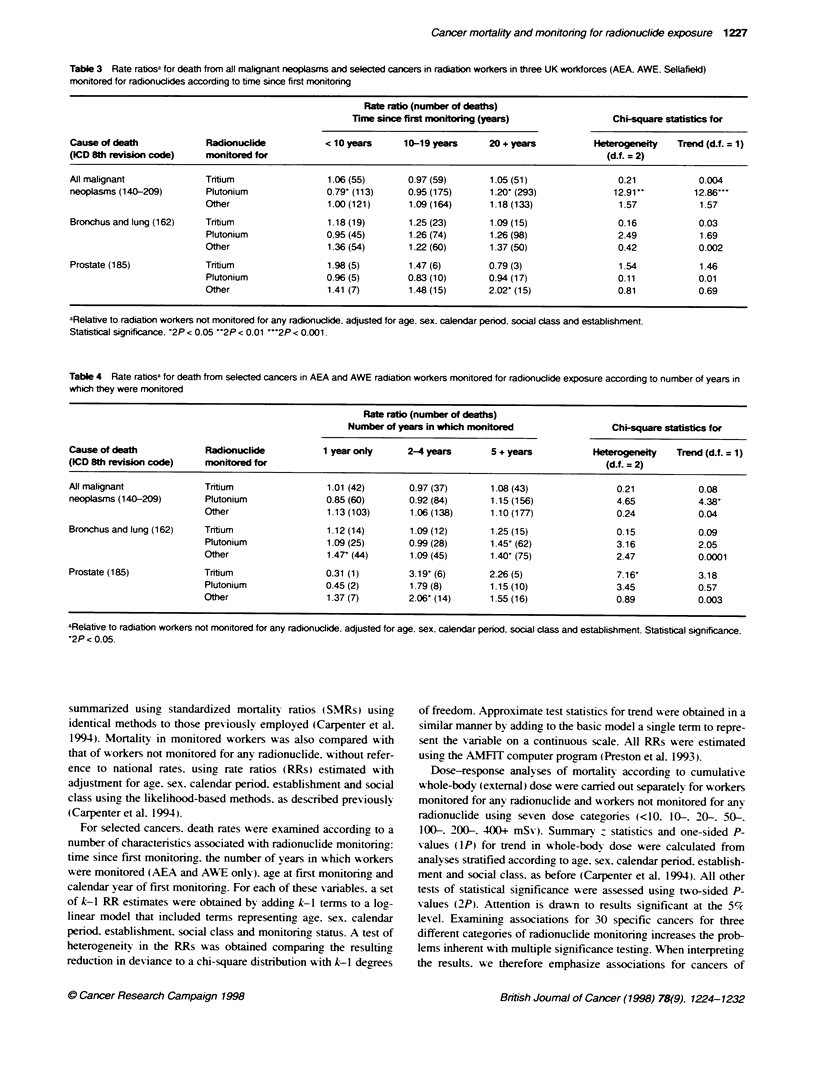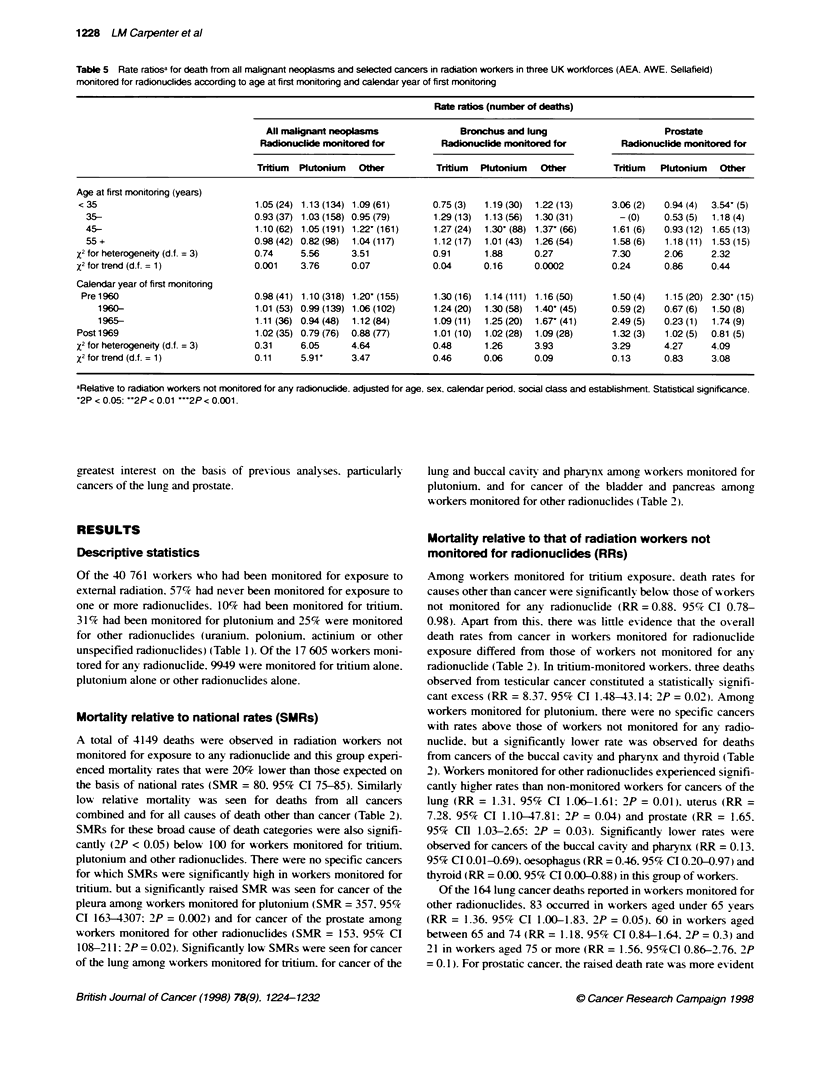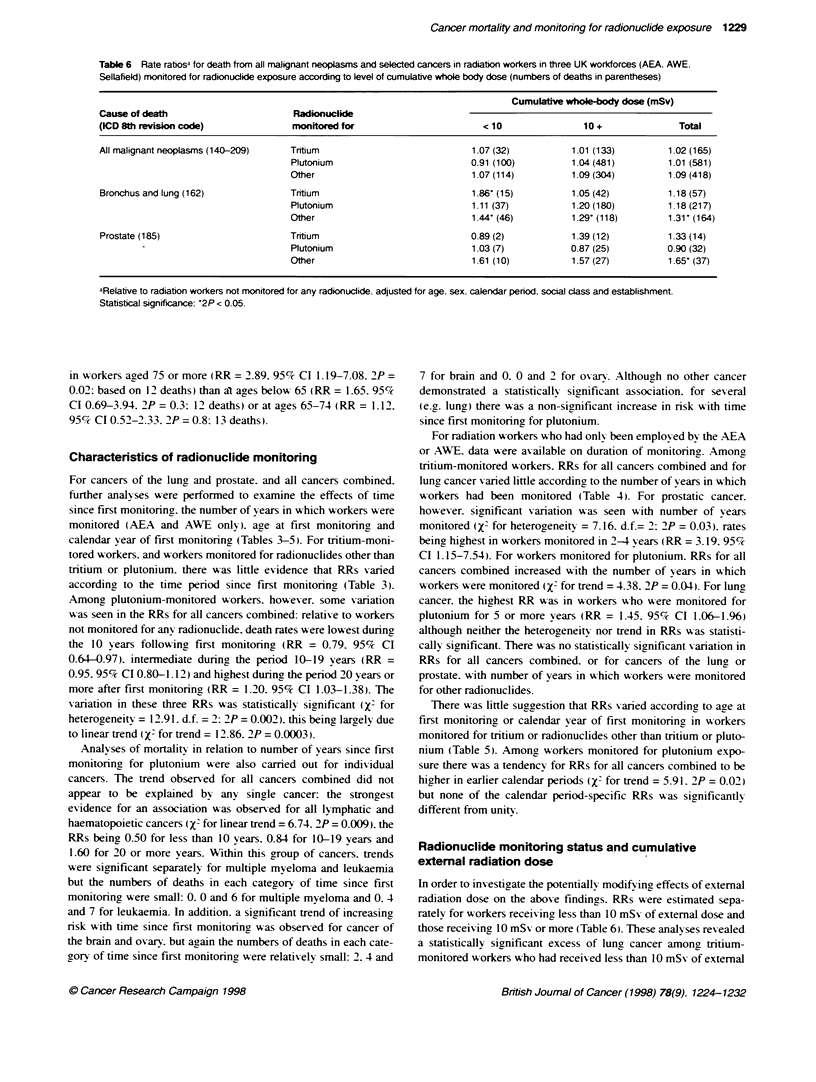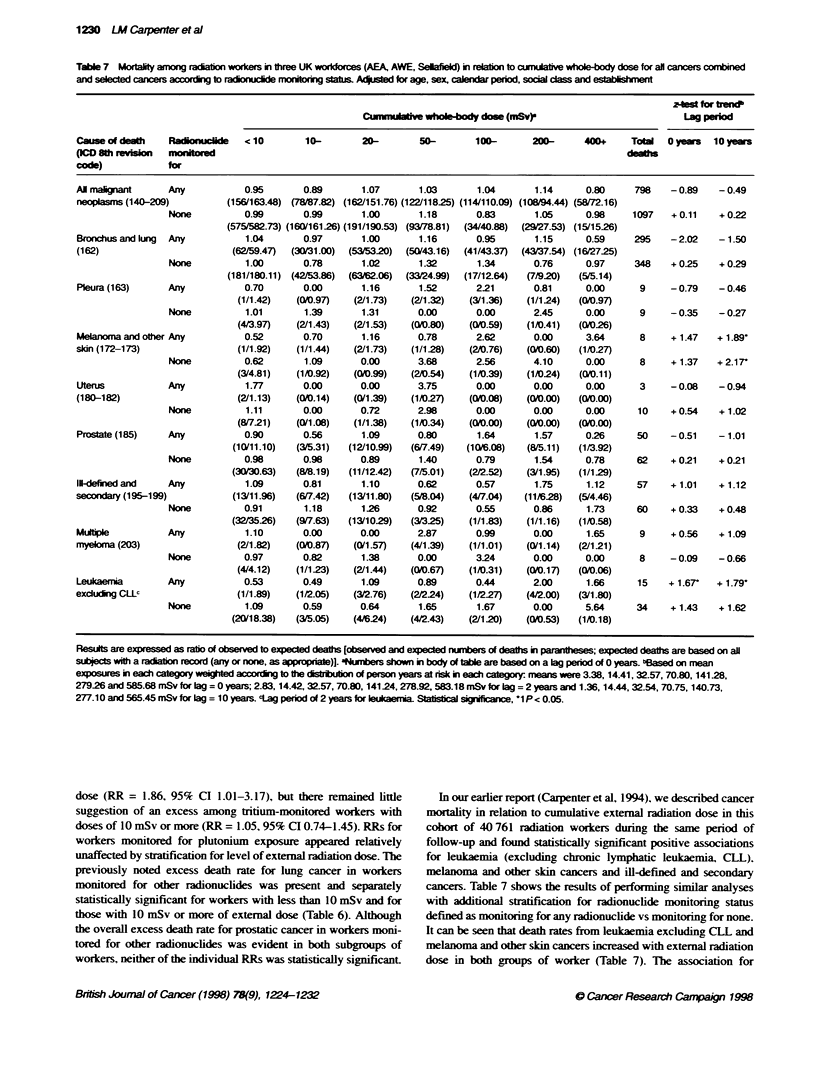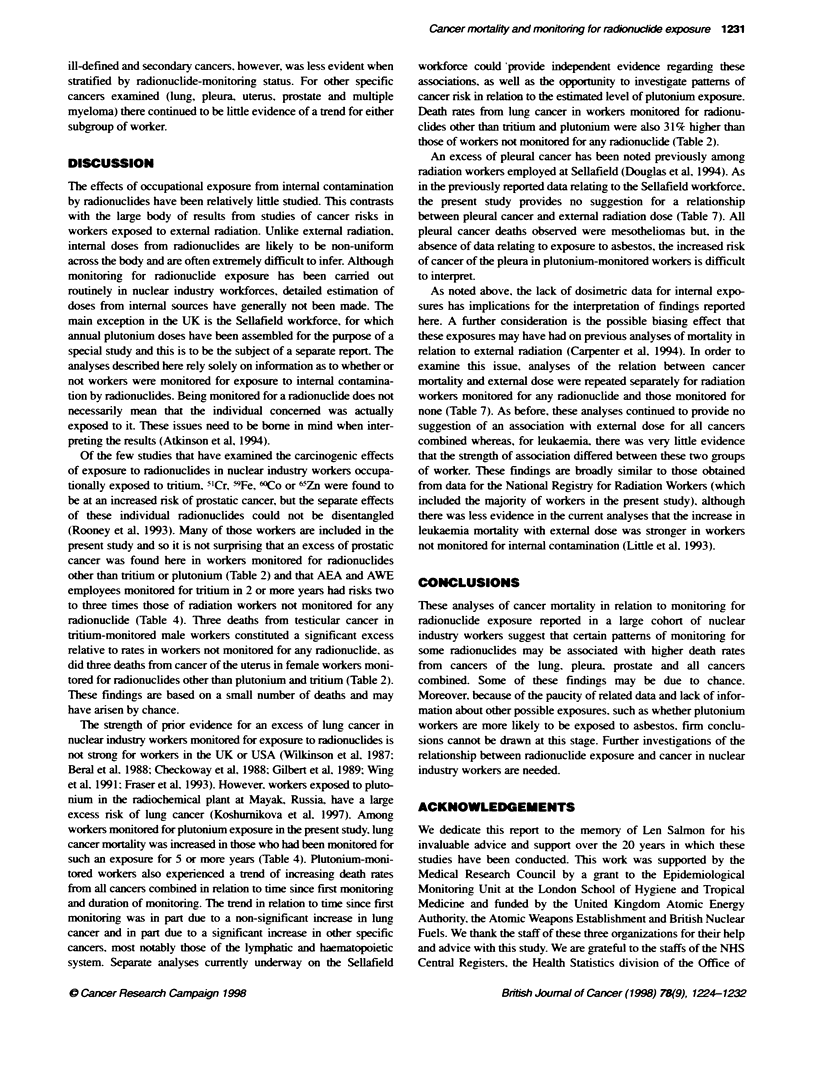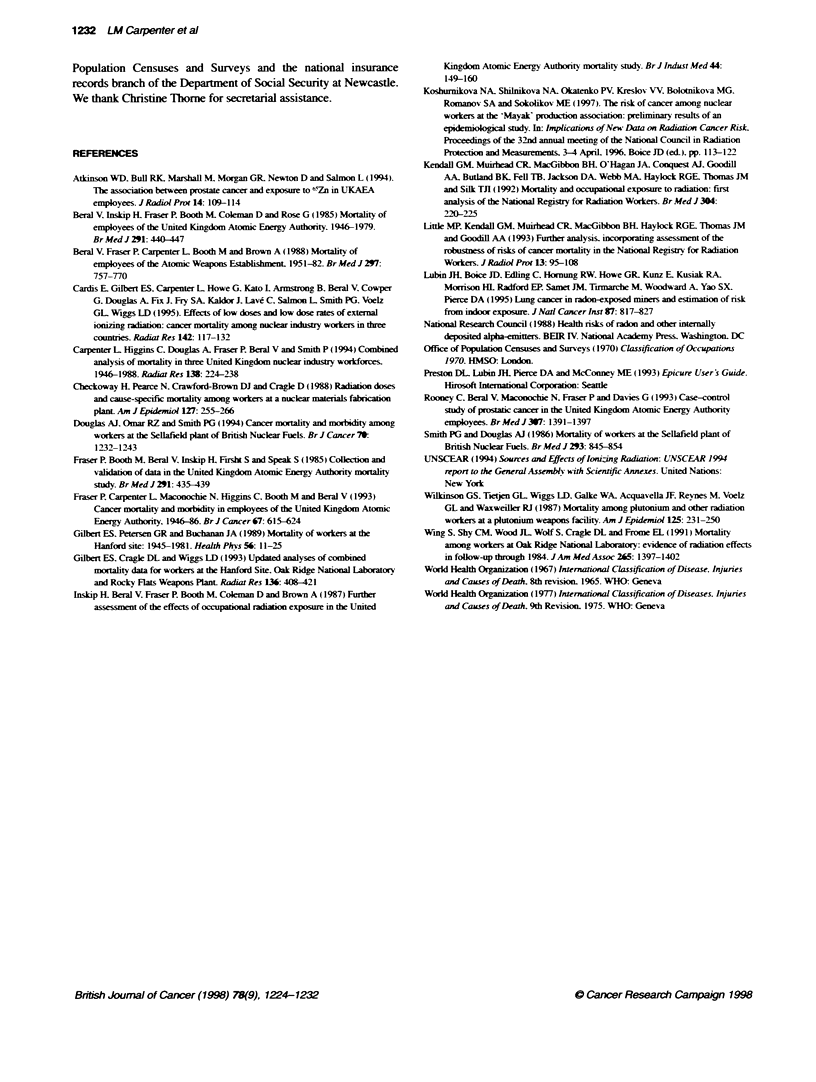# Cancer mortality in relation to monitoring for radionuclide exposure in three UK nuclear industry workforces.

**DOI:** 10.1038/bjc.1998.659

**Published:** 1998-11

**Authors:** L. M. Carpenter, C. D. Higgins, A. J. Douglas, N. E. Maconochie, R. Z. Omar, P. Fraser, V. Beral, P. G. Smith

**Affiliations:** Department of Public Health and Primary Care, University of Oxford, Radcliffe Infirmary, UK.

## Abstract

Cancer mortality in 40,761 employees of three UK nuclear industry facilities who had been monitored for external radiation exposure was examined according to whether they had also been monitored for possible internal exposure to tritium, plutonium or other radionuclides (uranium, polonium, actinium or other unspecified). Death rates from cancer were compared both with national rates and with rates in radiation workers not monitored for exposure to any radionuclides. Among workers monitored for tritium exposure, overall cancer mortality was significantly below national rates [standardized mortality ratio (SMR) = 83, 165 deaths; 2P = 0.02] and none of the cancer-specific death rates was significantly above either the national average or rates in non-monitored workers. Although the overall death rate from cancer in workers monitored for plutonium exposure was also significantly low relative to national rates (SMR = 89, 581 deaths; 2P = 0.005), mortality from pleural cancer was significantly raised (SMR = 357, nine deaths; 2P = 0.002); none of the rates differed significantly from those of non-monitored workers. Workers monitored for radionuclides other than tritium or plutonium also had a death rate from all cancers combined that was below the national average (SMR = 86, 418 deaths; 2P = 0.002) but prostatic cancer mortality was raised both in relation to death rates in the general population (SMR = 153, 37 deaths; 2P = 0.02) and to death rates in radiation workers who had not been monitored for exposure to any radionuclide [rate ratio (RR) = 1.65; 2P = 0.03]. Mortality from cancer of the lung was also significantly increased in workers monitored for other radionuclides compared with those of radiation workers not monitored for exposure to radionuclides (RR = 1.31, 164 deaths; 2P = 0.01). For cancers of the lung, prostate and all cancers combined, death rates in monitored workers were examined according to the timing and duration of monitoring for radionuclide exposure, with rates of radiation workers not monitored for any radionuclide forming the comparison group. In tritium-monitored workers, RRs for prostatic cancer varied significantly according to the number of years in which they were monitored (2P = 0.03). In workers monitored for plutonium exposure, RRs for all cancers combined increased with the number of years in which they were monitored (2P = 0.04) and with the number of years since first monitoring (2P = 0.0003). There was little suggestion of systematic variation in RRs for workers monitored for other radionuclides in relation to the timing or duration of monitoring, nor did it appear that their raised rates of cancer of the lung and prostate were explained by external radiation dose. These analyses of cancer mortality in relation to monitoring for radionuclide exposure reported in a large cohort of nuclear industry workers suggest that certain patterns of monitoring for some radionuclides may be associated with higher death rates from cancers of the lung, pleura, prostate and all cancers combined. Some of these findings may be due to chance. Moreover, because of the paucity of related data and lack of information about other possible exposures, such as whether plutonium workers are more likely to be exposed to asbestos, firm conclusions cannot be drawn at this stage. Further investigations of the relationship between radionuclide exposure and cancer in nuclear industry workers are needed.


British Journal of Cancer (1998) 78(9), 1224–1232
© 1998 Cancer Research Campaign

### Cancer mortality in relation to monitoring for radionuclide exposure in three UK nuclear industry workforces

**LM Carpenter[1], CD Higgins[2], AJ Douglas[2], NES Maconochie[2], RZ Omar[2], P Fraser[2], V Beral[3] and PG Smith[2]**

[1]Department of Public Health and Primary Care, University of Oxford, Gibson Building, Radcliffe Infirmary, Oxford OX2 6HE; [2]Department of Epidemiology and Population Sciences, London School of Hygiene and Tropical Medicine, Keppel Street, London WC1E 7HT; [3]Imperial Cancer Research Fund, Gibson Building, Radcliffe Infirmary, Oxford OX2 6HE, UK

**Summary** Cancer mortality in 40 761 employees of three UK nuclear industry facilities who had been monitored for external radiation exposure was examined according to whether they had also been monitored for possible internal exposure to tritium, plutonium or other radionuclides (uranium, polonium, actinium or other unspecified). Death rates from cancer were compared both with national rates and with rates in radiation workers not monitored for exposure to any radionuclides. Among workers monitored for tritium exposure, overall cancer mortality was significantly below national rates [standardized mortality ratio (SMR) = 83, 165 deaths; 2*P* = 0.02] and none of the cancer-specific death rates was significantly above either the national average or rates in non-monitored workers. Although the overall death rate from cancer in workers monitored for plutonium exposure was also significantly low relative to national rates (SMR = 89, 581 deaths; 2*P* = 0.005), mortality from pleural cancer was significantly raised (SMR = 357, nine deaths; 2*P* = 0.002); none of the rates differed significantly from those of non-monitored workers. Workers monitored for radionuclides other than tritium or plutonium also had a death rate from all cancers combined that was below the national average (SMR = 86, 418 deaths; 2*P* = 0.002) but prostatic cancer mortality was raised both in relation to death rates in the general population (SMR = 153, 37 deaths; 2*P* = 0.02) and to death rates in radiation workers who had not been monitored for exposure to any radionuclide [rate ratio (RR) = 1.65; 2*P* = 0.03]. Mortality from cancer of the lung was also significantly increased in workers monitored for other radionuclides compared with those of radiation workers not monitored for exposure to radionuclides (RR = 1.31, 164 deaths; 2*P* = 0.01). For cancers of the lung, prostate and all cancers combined, death rates in monitored workers were examined according to the timing and duration of monitoring for radionuclide exposure, with rates of radiation workers not monitored for any radionuclide forming the comparison group. In tritium-monitored workers, RRs for prostatic cancer varied significantly according to the number of years in which they were monitored (2*P* = 0.03). In workers monitored for plutonium exposure, RRs for all cancers combined increased with the number of years in which they were monitored (2*P* = 0.04) and with the number of years since first monitoring (2*P* = 0.0003). There was little suggestion of systematic variation in RRs for workers monitored for other radionuclides in relation to the timing or duration of monitoring, nor did it appear that their raised rates of cancer of the lung and prostate were explained by external radiation dose. These analyses of cancer mortality in relation to monitoring for radionuclide exposure reported in a large cohort of nuclear industry workers suggest that certain patterns of monitoring for some radionuclides may be associated with higher death rates from cancers of the lung, pleura, prostate and all cancers combined. Some of these findings may be due to chance. Moreover, because of the paucity of related data and lack of information about other possible exposures, such as whether plutonium workers are more likely to be exposed to asbestos, firm conclusions cannot be drawn at this stage. Further investigations of the relationship between radionuclide exposure and cancer in nuclear industry workers are needed.

**Keywords:** cancer mortality; nuclear industry workers; radionuclide monitoring; internal radiation exposure

Several large epidemiological studies have examined cancer mortality in nuclear industry workers in relation to occupational exposure to external ionizing radiation, and combined analyses of mortality have been published recently for the UK (Kendall et al, 1992; Carpenter et al, 1994) and for the US and Canada (Gilbert et al, 1993; Cardis et al, 1995). These studies have provided detailed information about the relationship between cancer risk in populations occupationally exposed to low-level ionizing radiation from external sources. The effects of occupational exposure to internal sources of radiation from radionuclides (such as tritium or plutonium) have, however, been little studied. These exposures occur in work environments where there are unsealed sources of radioactive material, when particles may enter the body by inhalation, ingestion or accidentally through a wound.

Excess risks of lung cancer have been documented in miners exposed to α-particle-emitting radon progeny (Lubin et al, 1995), but the effects of internal exposures typically found in the nuclear industry are less certain. Increased risks of bone and head and neck cancers have been associated with occupational exposure to radium and of lung cancer associated with occupational exposure to plutonium (Wilkinson et al, 1987; Checkoway et al, 1988; National Research Council, 1988; UNSCEAR, 1994; Koshurnikova et al, 1997). Our previous analyses of mortality in employees of the UK Atomic Energy Authority (AEA) and the

*Received 16 April 1997*
*Revised 16 February 1998*
*Accepted 11 March 1998*

*Correspondence to:* LM Carpenter

**Table 1** Numbers (per cent) of radiation workers in three UK workforces (AEA, AWE, Sellafield) according to their radionuclide monitoring status and sex

| | Total number of radiation workers | Not monitored for any radionuclide | Ever monitored for[a] | | |
|---|---|---|---|---|---|
| | | | Tritium | Plutonium | Other radionuclides |
| Male | 37 395 (100%) | 20 521 (54.9%) | 3 986 (10.7%) | 11 942 (31.9%) | 9 772 (26.1%) |
| Female | 3 366 (100%) | 2 635 (78.3%) | 125 (3.7%) | 556 (16.5%) | 413 (12.3%) |
| Total | 40 761 (100%) | 23 156 (56.8%) | 4 111 (10.1%) | 12 498 (30.7%) | 10 185 (25.0%) |

[a]Groups not mutually exclusive: of 17 605 workers monitored for any radionuclide, 9949 were monitored for tritium alone, plutonium alone or other radionuclides alone.

Atomic Weapons Establishment (AWE) suggested that workers who had been monitored for radionuclides were at increased risk of cancer of the prostate and possibly also of lung cancer (Beral et al, 1988; Fraser et al, 1993). The association with prostatic cancer was investigated in detail in a nested case–control study of employees of the AEA (Rooney et al, 1993). This revealed increased risks among workers occupationally exposed to tritium, $^{51}Cr$, $^{59}Fe$, $^{60}Co$ or $^{65}Zn$, but the effects of these individual radionuclides could not be disentangled.

We have previously reported combined analyses of cancer mortality in relation to exposure to external radiation for three UK cohorts comprising employees of the AEA, the AWE and the Sellafield plant of British Nuclear Fuels Limited (BNFL) (Carpenter et al, 1994). In the present report we examine cancer mortality of workers in these three cohorts who had been monitored for exposure to external radiation according to whether or not they were also monitored for internal exposure to tritium or plutonium and (for AEA and AWE employees only) to other radionuclides (uranium, polonium, actinium or other unspecified). Particular attention is given in the analyses to mortality from cancers of the lung and prostate because these cancers have been associated with the specific type of radionuclides to which nuclear industry workers are exposed.

### MATERIALS AND METHODS

### Study population and personnel data

The study population derives from the combined cohort of 75 006 employees that formed the basis of our previous report (Carpenter et al, 1994). This comprised all individuals who had worked at the AEA establishments at Harwell (with Culham and London), Dounreay or Winfrith before 1980, at the AWE before 1983 or at Sellafield before 1976. The present analyses relate to the subset of 40 761 monitored workers for whom personal dose records had been maintained by one or more of the contributing establishments. Details are provided elsewhere of the methods used for data collection and validation separately for each contributing cohort (Beral et al, 1985, 1988; Fraser et al, 1985, 1993; Smith and Douglas, 1986; Douglas et al, 1994; Inskip et al, 1987) and for the assembly of data for the combined study (Carpenter et al, 1994).

### Mortality data

Deaths and emigrations reported in cohort members by the National Health Service Central Registers (NHSCRs) up to the end of 1988 were included, as in our previous analyses (Carpenter et al, 1994). All analyses were based on the underlying cause of death (as stated on the death certificate) coded according to the International Classification of Diseases (ICD) (World Health Organization, 1967, 1977). Deaths for which an underlying cause could not be ascertained were included in analyses of death from all causes but not in cause-specific analyses. The present analyses are based on a total of 6944 deaths in workers monitored for radiation exposure, of which 1895 were from cancer. This includes 11 additional cancer deaths identified following the introduction of the new computerized system at NHSCR in Southport that were not available for inclusion in our previous analyses (Carpenter et al, 1994).

### Radiation data

For AEA and AWE employees, information on annual monitoring of personnel for possible intake of radionuclides [tritium, plutonium and other radionuclides (uranium, polonium, actinium and other unspecified)] was provided in the form of a set of annual flags for each worker, indicating for each year whether they had been monitored for each radionuclide or group of radionuclides. For Sellafield workers, information on radionuclide monitoring was available for plutonium and tritium only and was limited to the year in which the worker was first monitored for each radionuclide. There was insufficient detail about radionuclides other than tritium or plutonium to warrant separate analysis. Data on external radiation dose were obtained from records held by the three industries, and the methods used in their assembly have been described in detail previously (Beral et al, 1985, 1988; Fraser et al, 1985, 1993; Smith and Douglas, 1986; Inskip et al, 1987; Douglas et al, 1994). For the majority of the study period, regulatory dose records did not generally include doses to organs from the intake of radioactivity (internal dose) (Carpenter et al, 1994).

### Statistical methods

Workers contributed person–years (PY) at risk from their earliest date of first monitoring for radiation at AEA, AWE or Sellafield through to 31 December 1988 or their date of emigration, date of death or the date they were last traced, if any of these preceded 1 January 1989. PY at risk and deaths were stratified by sex, age in 15 groups (15–, 20–, ... 85+ years), calendar year in single years (for comparisons with national rates) and in nine groups (1946–, 1950–, 1955–, ... 1985–88, for all other analyses), last establishment in five groups (Harwell with Culham and London, Dounreay, Winfrith, AWE, Sellafield) and social class in up to four groups (I+II, III non-manual, III manual and IV+V coded according to the British Registrar General's Classification (Office of Population Censuses and Surveys, 1970) for AEA and AWE; non-industrial

© *Cancer Research Campaign 1998*

**Table 2** Standardized mortality ratios[a] (SMRs) and rate ratios[b] (RRs) for specific cancers in radiation workers in three UK workforces (AEA, AWE, Sellafield) according to their radionuclide monitoring status

| Cause of death (ICD 8th revision code) | Not monitored for any radionuclide | Ever monitored for: Tritium | | Ever monitored for: Plutonium | | Ever monitored for: Other radionuclides | |
|---|---|---|---|---|---|---|---|
| | SMR (observed deaths) | SMR (observed deaths) | RR (95% CI) | SMR (observed deaths) | RR (95% CI) | SMR (observed deaths) | RR (95% CI) |
| All malignant neoplasms (140–209) | 80*** (1097) | 83* (165) | 1.02 (0.86–1.21) | 89** (581) | 1.01 (0.90–1.13) | 86** (418) | 1.09 (0.96–1.23) |
| Buccal cavity and pharynx (140–149) | 88 (18) | – (0) | 0.0* (0.0–0.90) | 30*(3) | 0.29*(0.07–0.94) | 13 (1) | 0.13*(0.01–0.69) |
| Oesophagus (150) | 89 (37) | 88 (6) | 0.86 (0.32–1.97) | 108 (23) | 0.81 (0.46–1.39) | 56 (9) | 0.46* (0.20–0.97) |
| Stomach (151) | 87 (114) | 62 (11) | 0.68 (0.34–1.23) | 89 (54) | 0.85 (0.60–1.21) | 79 (35) | 1.04 (0.67–1.57) |
| Small intestine (152) | 110 (3) | 249 (1) | 1.10 (0.05–10.69) | 307 (4) | 2.31 (0.46–13.45) | 207 (2) | 1.00 (0.13–6.47) |
| Large intestine (153) | 96 (84) | 81 (10) | 0.75 (0.36–1.41) | 94 (38) | 0.84 (0.55–1.26) | 79 (24) | 0.81 (0.49–1.31) |
| Rectum (154) | 75 (46) | 57 (5) | 0.74 (0.25–1.77) | 87 (25) | 1.02 (0.59–1.75) | 70 (15) | 0.93 (0.48–1.73) |
| Liver and gall bladder (155–156) | 51* (9) | 111 (3) | 2.32 (0.43–9.99) | 70 (6) | 2.00 (0.59–6.38) | 77 (5) | 1.16 (0.32–3.74) |
| Pancreas (157) | 82 (47) | 92 (8) | 1.09 (0.46–2.24) | 68 (19) | 0.72 (0.40–1.27) | 48* (10) | 0.53 (0.24–1.05) |
| Nasal cavities and sinuses (160) | 108 (3) | 246 (1) | 1.33 (0.06–12.53) | 151 (2) | 1.23 (0.14–9.18) | 203 (2) | 2.25 (0.24–20.47) |
| Larynx (161) | 48 (6) | 53 (1) | 2.10 (0.10–15.58) | 65 (4) | 2.55 (0.58–10.87) | 44 (2) | 0.97 (0.13–5.07) |
| Bronchus and lung (162) | 68*** (348) | 73* (57) | 1.18 (0.87–1.58) | 85* (217) | 1.18 (0.97–1.42) | 86 (164) | 1.31* (1.06–1.61) |
| Pleura (163) | 209 (9) | 115 (1) | 0.48 (0.03–2.69) | 357** (9) | 1.97 (0.71–5.49) | 200 (4) | 1.62 (0.38–6.27) |
| Bone (170) | 83 (4) | 181 (1) | 1.31 (0.05–14.71) | 100 (2) | 1.01 (0.12–7.35) | 142 (2) | 2.07 (0.21–18.61) |
| Connective tissue (171) | 111 (5) | – (0) | 0.0 (0.0–4.57) | 45 (1) | 0.35 (0.02–2.43) | 60 (1) | 1.27 (0.06–12.63) |
| Melanoma and other skin (172–173) | 58 (8) | 148 (3) | 1.50 (0.31–5.78) | 46 (3) | 0.50 (0.11–1.88) | 102 (5) | 1.76 (0.42–7.32) |
| Breast (174–175) | 64 (16) | – (0) | 0.0 (0.0–2.31) | 117 (5) | 2.17 (0.63–6.70) | 33 (1) | 0.54 (0.03–3.05) |
| All female genital (180–184) | 121 (18) | 203 (1) | 1.95 (0.10–13.04) | 189 (4) | 2.14 (0.52–7.78) | 201 (3) | 2.56 (0.52–9.57) |
| Uterus (180–182) | 144 (10) | 442 (1) | 2.99 (0.13–30.03) | 203 (2) | 1.67 (0.22–9.63) | 432 (3) | 7.28* (1.10–47.81) |
| Ovary (183) | 95 (7) | – (0) | 0.0 (0.0–34.49) | 186 (2) | 4.88 (0.49–48.44) | – (0) | 0.0 (0.0–6.42) |
| All male genital (185–187) | 89 (69) | 164 (17) | 1.61 (0.88–2.79) | 102 (36) | 0.99 (0.63–1.53) | 142 (38) | 1.56 (0.99–2.47) |
| Prostate (185) | 90 (62) | 150 (14) | 1.33 (0.69–2.41) | 101 (32) | 0.90 (0.56–1.43) | 153* (37) | 1.65* (1.03–2.65) |
| Testis (186) | 111 (7) | 401 (3) | 8.37* (1.48–43.14) | 152 (4) | 2.36 (0.55–8.91) | 53 (1) | 0.60 (0.03–4.12) |
| Bladder (188) | 90 (44) | 116 (8) | 1.51 (0.64–3.17) | 65 (15) | 0.72 (0.37–1.34) | 46* (8) | 0.56 (0.23–1.19) |
| Kidney (189.0) | 89 (21) | 78 (3) | 0.65 (0.15–1.97) | 100 (12) | 0.89 (0.41–1.87) | 110 (10) | 1.53 (0.63–3.57) |
| Brain and other central nervous system[c] (191–192, 225, 238) | 79 (36) | 86 (6) | 1.08 (0.40–2.48) | 76 (17) | 0.89 (0.46–1.66) | 85 (14) | 1.26 (0.61–2.51) |
| Thyroid (193) | 269* (7) | – (0) | 0.0 (0.0–2.50) | 85 (1) | 0.15* (0.01–0.89) | – (0) | 0.0* (0.0–0.88) |
| Ill-defined and secondary (195–199) | 95 (60) | 100 (10) | 0.93 (0.43–1.82) | 121 (38) | 1.11 (0.71–1.72) | 137 (33) | 1.50 (0.92–2.41) |
| All lymphatic and haematopoietic (200–209) | 93 (87) | 88 (12) | 1.03 (0.52–1.87) | 92 (41) | 1.04 (0.69–1.56) | 87 (29) | 0.85 (0.53–1.33) |
| Non-Hodgkin's lymphoma (200, 202) | 109 (29) | 167 (7) | 1.90 (0.74–4.30) | 129 (17) | 1.48 (0.76–2.83) | 120 (12) | 0.90 (0.43–1.81) |
| Hodgkin's disease (201) | 55 (7) | 65 (1) | 0.94 (0.05–6.16) | 110 (6) | 1.44 (0.41–5.16) | 79 (3) | 1.02 (0.19–4.73) |
| Multiple myeloma (203) | 53 (8) | 42 (1) | 0.63 (0.03–3.74) | 79 (6) | 1.05 (0.33–3.21) | 87 (5) | 1.98 (0.53–7.28) |
| Leukaemia (204–208) | 117 (43) | 59 (3) | 0.55 (0.13–1.59) | 65 (11) | 0.62 (0.29–1.23) | 72 (9) | 0.58 (0.25–1.22) |
| Leukaemia excluding CLL | –[d] (34) | –[d] (3) | 0.66 (0.15–1.98) | –[d] (9) | 0.58 (0.25–1.24) | –[d] (7) | 0.56 (0.21–1.29) |
| *Causes other than cancer (0–139, 210–999)* | 80*** (3052) | 72*** (363) | 0.88* (0.78–0.98) | 88*** (1508) | 0.97 (0.91–1.04) | 76*** (967) | 0.94 (0.86–1.01) |
| *All causes* (0–999) | 80*** (4149) | 75*** (528) | 0.92 (0.83–1.01) | 88*** (2089) | 0.98 (0.93–1.04) | 79*** (1385) | 0.98 (0.91–1.05) |
| Number of workers monitored | 23 156 | 4111 | | 12 498 | | 10 185 | |

[a]Using age-, sex-, and calendar year-specific rates in England and Wales. [b]Relative to radiation workers not monitored for any radionuclide, adjusted for age, sex, calendar period, social class and establishment. [c]Includes benign and unspecified neoplasms of nervous system. [d]Rates for England and Wales not available. Significance of difference from 100 (SMR) or 1 (RR), *2$P$ < 0.05; **2$P$ < 0.01; ***2$P$ < 0.001.

and industrial for Sellafield). Ten employees missing vital information (e.g. date of birth) were excluded from all analyses and 36 employees who could not be traced at the NHSCRs contributed PY at risk until their date of last employment.

Radionuclide monitoring status was treated as a time-dependent variable in all analyses. For example, a plutonium-monitored worker's PY at risk were added to those of workers not monitored for any radionuclide up to the date in which they were first monitored for plutonium, after which their PY at risk (and death, if applicable) were added to the plutonium-monitored group. Similarly, for time-dependent variables such as time since first monitoring (classified in three levels: < 10 years, 10–19 years and 20+ years) workers contributed PY at risk to the first category until their tenth anniversary of first monitoring, after which they contributed to the second category and so on.

For radionuclide-monitored workers, and those not monitored for any radionuclide, age, sex and calender year-specific death rates were assessed relative to those for England and Wales and

© Cancer Research Campaign 1998

**Table 3** Rate ratios[a] for death from all malignant neoplasms and selected cancers in radiation workers in three UK workforces (AEA, AWE, Sellafield) monitored for radionuclides according to time since first monitoring

| Cause of death (ICD 8th revision code) | Radionuclide monitored for | Rate ratio (number of deaths) Time since first monitoring (years): < 10 years | 10–19 years | 20 + years | Chi-square statistics for: Heterogeneity (d.f. = 2) | Trend (d.f. = 1) |
|---|---|---|---|---|---|---|
| All malignant neoplasms (140–209) | Tritium | 1.06 (55) | 0.97 (59) | 1.05 (51) | 0.21 | 0.004 |
| | Plutonium | 0.79* (113) | 0.95 (175) | 1.20* (293) | 12.91** | 12.86*** |
| | Other | 1.00 (121) | 1.09 (164) | 1.18 (133) | 1.57 | 1.57 |
| Bronchus and lung (162) | Tritium | 1.18 (19) | 1.25 (23) | 1.09 (15) | 0.16 | 0.03 |
| | Plutonium | 0.95 (45) | 1.26 (74) | 1.26 (98) | 2.49 | 1.69 |
| | Other | 1.36 (54) | 1.22 (60) | 1.37 (50) | 0.42 | 0.002 |
| Prostate (185) | Tritium | 1.98 (5) | 1.47 (6) | 0.79 (3) | 1.54 | 1.46 |
| | Plutonium | 0.96 (5) | 0.83 (10) | 0.94 (17) | 0.11 | 0.01 |
| | Other | 1.41 (7) | 1.48 (15) | 2.02* (15) | 0.81 | 0.69 |

[a]Relative to radiation workers not monitored for any radionuclide, adjusted for age, sex, calendar period, social class and establishment.
Statistical significance, *2$P$ < 0.05 **2$P$ < 0.01 ***2$P$ < 0.001.

**Table 4** Rate ratios[a] for death from selected cancers in AEA and AWE radiation workers monitored for radionuclide exposure according to number of years in which they were monitored

| Cause of death (ICD 8th revision code) | Radionuclide monitored for | Rate ratio (number of deaths) Number of years in which monitored: 1 year only | 2–4 years | 5 + years | Chi-square statistics for: Heterogeneity (d.f. = 2) | Trend (d.f. = 1) |
|---|---|---|---|---|---|---|
| All malignant neoplasms (140–209) | Tritium | 1.01 (42) | 0.97 (37) | 1.08 (43) | 0.21 | 0.08 |
| | Plutonium | 0.85 (60) | 0.92 (84) | 1.15 (156) | 4.65 | 4.38* |
| | Other | 1.13 (103) | 1.06 (138) | 1.10 (177) | 0.24 | 0.04 |
| Bronchus and lung (162) | Tritium | 1.12 (14) | 1.09 (12) | 1.25 (15) | 0.15 | 0.09 |
| | Plutonium | 1.09 (25) | 0.99 (28) | 1.45* (62) | 3.16 | 2.05 |
| | Other | 1.47* (44) | 1.09 (45) | 1.40* (75) | 2.47 | 0.0001 |
| Prostate (185) | Tritium | 0.31 (1) | 3.19* (6) | 2.26 (5) | 7.16* | 3.18 |
| | Plutonium | 0.45 (2) | 1.79 (8) | 1.15 (10) | 3.45 | 0.57 |
| | Other | 1.37 (7) | 2.06* (14) | 1.55 (16) | 0.89 | 0.003 |

[a]Relative to radiation workers not monitored for any radionuclide, adjusted for age, sex, calendar period, social class and establishment. Statistical significance, *2$P$ < 0.05.

summarized using standardized mortality ratios (SMRs) using identical methods to those previously employed (Carpenter et al, 1994). Mortality in monitored workers was also compared with that of workers not monitored for any radionuclide, without reference to national rates, using rate ratios (RRs) estimated with adjustment for age, sex, calendar period, establishment and social class using the likelihood-based methods, as described previously (Carpenter et al, 1994).

For selected cancers, death rates were examined according to a number of characteristics associated with radionuclide monitoring: time since first monitoring, the number of years in which workers were monitored (AEA and AWE only), age at first monitoring and calendar year of first monitoring. For each of these variables, a set of $k$–1 RR estimates were obtained by adding $k$–1 terms to a log-linear model that included terms representing age, sex, calendar period, establishment, social class and monitoring status. A test of heterogeneity in the RRs was obtained comparing the resulting reduction in deviance to a chi-square distribution with $k$–1 degrees of freedom. Approximate test statistics for trend were obtained in a similar manner by adding to the basic model a single term to represent the variable on a continuous scale. All RRs were estimated using the AMFIT computer program (Preston et al, 1993).

Dose–response analyses of mortality according to cumulative whole-body (external) dose were carried out separately for workers monitored for any radionuclide and workers not monitored for any radionuclide using seven dose categories (<10, 10–, 20–, 50–, 100–, 200–, 400+ mSv). Summary $z$ statistics and one-sided $P$-values (1$P$) for trend in whole-body dose were calculated from analyses stratified according to age, sex, calendar period, establishment and social class, as before (Carpenter et al, 1994). All other tests of statistical significance were assessed using two-sided $P$-values (2$P$). Attention is drawn to results significant at the 5% level. Examining associations for 30 specific cancers for three different categories of radionuclide monitoring increases the problems inherent with multiple significance testing. When interpreting the results, we therefore emphasize associations for cancers of

© Cancer Research Campaign 1998 

**Table 5** Rate ratios[a] for death from all malignant neoplasms and selected cancers in radiation workers in three UK workforces (AEA, AWE, Sellafield) monitored for radionuclides according to age at first monitoring and calendar year of first monitoring

| | Rate ratios (number of deaths) | | | | | | | | |
|---|---|---|---|---|---|---|---|---|---|
| | All malignant neoplasms Radionuclide monitored for | | | Bronchus and lung Radionuclide monitored for | | | Prostate Radionuclide monitored for | | |
| | Tritium | Plutonium | Other | Tritium | Plutonium | Other | Tritium | Plutonium | Other |
| Age at first monitoring (years) | | | | | | | | | |
| < 35 | 1.05 (24) | 1.13 (134) | 1.09 (61) | 0.75 (3) | 1.19 (30) | 1.22 (13) | 3.06 (2) | 0.94 (4) | 3.54* (5) |
| 35– | 0.93 (37) | 1.03 (158) | 0.95 (79) | 1.29 (13) | 1.13 (56) | 1.30 (31) | – (0) | 0.53 (5) | 1.18 (4) |
| 45– | 1.10 (62) | 1.05 (191) | 1.22* (161) | 1.27 (24) | 1.30* (88) | 1.37* (66) | 1.61 (6) | 0.93 (12) | 1.65 (13) |
| 55 + | 0.98 (42) | 0.82 (98) | 1.04 (117) | 1.12 (17) | 1.01 (43) | 1.26 (54) | 1.58 (6) | 1.18 (11) | 1.53 (15) |
| $\chi^2$ for heterogeneity (d.f. = 3) | 0.74 | 5.56 | 3.51 | 0.91 | 1.88 | 0.27 | 7.30 | 2.06 | 2.32 |
| $\chi^2$ for trend (d.f. = 1) | 0.001 | 3.76 | 0.07 | 0.04 | 0.16 | 0.0002 | 0.24 | 0.86 | 0.44 |
| Calendar year of first monitoring | | | | | | | | | |
| Pre 1960 | 0.98 (41) | 1.10 (318) | 1.20* (155) | 1.30 (16) | 1.14 (111) | 1.16 (50) | 1.50 (4) | 1.15 (20) | 2.30* (15) |
| 1960– | 1.01 (53) | 0.99 (139) | 1.06 (102) | 1.24 (20) | 1.30 (58) | 1.40* (45) | 0.59 (2) | 0.67 (6) | 1.50 (8) |
| 1965– | 1.11 (36) | 0.94 (48) | 1.12 (84) | 1.09 (11) | 1.25 (20) | 1.67* (41) | 2.49 (5) | 0.23 (1) | 1.74 (9) |
| Post 1969 | 1.02 (35) | 0.79 (76) | 0.88 (77) | 1.01 (10) | 1.02 (28) | 1.09 (28) | 1.32 (3) | 1.02 (5) | 0.81 (5) |
| $\chi^2$ for heterogeneity (d.f. = 3) | 0.31 | 6.05 | 4.64 | 0.48 | 1.26 | 3.93 | 3.29 | 4.27 | 4.09 |
| $\chi^2$ for trend (d.f. = 1) | 0.11 | 5.91* | 3.47 | 0.46 | 0.06 | 0.09 | 0.13 | 0.83 | 3.08 |

[a]Relative to radiation workers not monitored for any radionuclide, adjusted for age, sex, calendar period, social class and establishment. Statistical significance. *2P < 0.05; **2P < 0.01 ***2P < 0.001.

greatest interest on the basis of previous analyses, particularly cancers of the lung and prostate.

### RESULTS

### Descriptive statistics

Of the 40 761 workers who had been monitored for exposure to external radiation, 57% had never been monitored for exposure to one or more radionuclides, 10% had been monitored for tritium, 31% had been monitored for plutonium and 25% were monitored for other radionuclides (uranium, polonium, actinium or other unspecified radionuclides) (Table 1). Of the 17 605 workers monitored for any radionuclide, 9949 were monitored for tritium alone, plutonium alone or other radionuclides alone.

### Mortality relative to national rates (SMRs)

A total of 4149 deaths were observed in radiation workers not monitored for exposure to any radionuclide and this group experienced mortality rates that were 20% lower than those expected on the basis of national rates (SMR = 80, 95% CI 75–85). Similarly low relative mortality was seen for deaths from all cancers combined and for all causes of death other than cancer (Table 2). SMRs for these broad cause of death categories were also significantly ($2P < 0.05$) below 100 for workers monitored for tritium, plutonium and other radionuclides. There were no specific cancers for which SMRs were significantly high in workers monitored for tritium, but a significantly raised SMR was seen for cancer of the pleura among workers monitored for plutonium (SMR = 357, 95% CI 163–4307; $2P = 0.002$) and for cancer of the prostate among workers monitored for other radionuclides (SMR = 153, 95% CI 108–211; $2P = 0.02$). Significantly low SMRs were seen for cancer of the lung among workers monitored for tritium, for cancer of the lung and buccal cavity and pharynx among workers monitored for plutonium, and for cancer of the bladder and pancreas among workers monitored for other radionuclides (Table 2).

### Mortality relative to that of radiation workers not monitored for radionuclides (RRs)

Among workers monitored for tritium exposure, death rates for causes other than cancer were significantly below those of workers not monitored for any radionuclide (RR = 0.88, 95% CI 0.78–0.98). Apart from this, there was little evidence that the overall death rates from cancer in workers monitored for radionuclide exposure differed from those of workers not monitored for any radionuclide (Table 2). In tritium-monitored workers, three deaths observed from testicular cancer constituted a statistically significant excess (RR = 8.37, 95% CI 1.48–43.14; $2P = 0.02$). Among workers monitored for plutonium, there were no specific cancers with rates above those of workers not monitored for any radionuclide, but a significantly lower rate was observed for deaths from cancers of the buccal cavity and pharynx and thyroid (Table 2). Workers monitored for other radionuclides experienced significantly higher rates than non-monitored workers for cancers of the lung (RR = 1.31, 95% CI 1.06–1.61; $2P = 0.01$), uterus (RR = 7.28, 95% CI 1.10–47.81; $2P = 0.04$) and prostate (RR = 1.65, 95% CIl 1.03–2.65; $2P = 0.03$). Significantly lower rates were observed for cancers of the buccal cavity and pharynx (RR = 0.13, 95% CI 0.01–0.69), oesophagus (RR = 0.46, 95% CI 0.20–0.97) and thyroid (RR = 0.00, 95% CI 0.00–0.88) in this group of workers.

Of the 164 lung cancer deaths reported in workers monitored for other radionuclides, 83 occurred in workers aged under 65 years (RR = 1.36, 95% CI 1.00–1.83, $2P = 0.05$), 60 in workers aged between 65 and 74 (RR = 1.18, 95% CI 0.84–1.64, $2P = 0.3$) and 21 in workers aged 75 or more (RR = 1.56, 95%CI 0.86–2.76, $2P = 0.1$). For prostatic cancer, the raised death rate was more evident

 © Cancer Research Campaign 1998

**Table 6** Rate ratios[a] for death from all malignant neoplasms and selected cancers in radiation workers in three UK workforces (AEA. AWE. Sellafield) monitored for radionuclide exposure according to level of cumulative whole body dose (numbers of deaths in parentheses)

| Cause of death (ICD 8th revision code) | Radionuclide monitored for | Cumulative whole-body dose (mSv) < 10 | 10 + | Total |
|---|---|---|---|---|
| All malignant neoplasms (140–209) | Tritium | 1.07 (32) | 1.01 (133) | 1.02 (165) |
| | Plutonium | 0.91 (100) | 1.04 (481) | 1.01 (581) |
| | Other | 1.07 (114) | 1.09 (304) | 1.09 (418) |
| Bronchus and lung (162) | Tritium | 1.86* (15) | 1.05 (42) | 1.18 (57) |
| | Plutonium | 1.11 (37) | 1.20 (180) | 1.18 (217) |
| | Other | 1.44* (46) | 1.29* (118) | 1.31* (164) |
| Prostate (185) | Tritium | 0.89 (2) | 1.39 (12) | 1.33 (14) |
| | Plutonium | 1.03 (7) | 0.87 (25) | 0.90 (32) |
| | Other | 1.61 (10) | 1.57 (27) | 1.65* (37) |

[a]Relative to radiation workers not monitored for any radionuclide. adjusted for age. sex. calendar period. social class and establishment. Statistical significance: *2$P$ < 0.05.

in workers aged 75 or more (RR = 2.89. 95% CI 1.19–7.08. 2$P$ = 0.02: based on 12 deaths) than at ages below 65 (RR = 1.65. 95% CI 0.69–3.94. 2$P$ = 0.3: 12 deaths) or at ages 65–74 (RR = 1.12. 95% CI 0.52–2.33. 2$P$ = 0.8: 13 deaths).

### Characteristics of radionuclide monitoring

For cancers of the lung and prostate. and all cancers combined. further analyses were performed to examine the effects of time since first monitoring. the number of years in which workers were monitored (AEA and AWE only). age at first monitoring and calendar year of first monitoring (Tables 3–5). For tritium-monitored workers. and workers monitored for radionuclides other than tritium or plutonium. there was little evidence that RRs varied according to the time period since first monitoring (Table 3). Among plutonium-monitored workers. however. some variation was seen in the RRs for all cancers combined: relative to workers not monitored for any radionuclide. death rates were lowest during the 10 years following first monitoring (RR = 0.79. 95% CI 0.64–0.97). intermediate during the period 10–19 years (RR = 0.95. 95% CI 0.80–1.12) and highest during the period 20 years or more after first monitoring (RR = 1.20. 95% CI 1.03–1.38). The variation in these three RRs was statistically significant ($\chi^2$ for heterogeneity = 12.91. d.f. = 2: 2$P$ = 0.002). this being largely due to linear trend ($\chi^2$ for trend = 12.86. 2$P$ = 0.0003).

Analyses of mortality in relation to number of years since first monitoring for plutonium were also carried out for individual cancers. The trend observed for all cancers combined did not appear to be explained by any single cancer: the strongest evidence for an association was observed for all lymphatic and haematopoietic cancers ($\chi^2$ for linear trend = 6.74. 2$P$ = 0.009). the RRs being 0.50 for less than 10 years. 0.84 for 10–19 years and 1.60 for 20 or more years. Within this group of cancers. trends were significant separately for multiple myeloma and leukaemia but the numbers of deaths in each category of time since first monitoring were small: 0. 0 and 6 for multiple myeloma and 0. 4 and 7 for leukaemia. In addition. a significant trend of increasing risk with time since first monitoring was observed for cancer of the brain and ovary. but again the numbers of deaths in each category of time since first monitoring were relatively small: 2. 4 and 7 for brain and 0. 0 and 2 for ovary. Although no other cancer demonstrated a statistically significant association. for several (e.g. lung) there was a non-significant increase in risk with time since first monitoring for plutonium.

For radiation workers who had only been employed by the AEA or AWE. data were available on duration of monitoring. Among tritium-monitored workers. RRs for all cancers combined and for lung cancer varied little according to the number of years in which workers had been monitored (Table 4). For prostatic cancer. however. significant variation was seen with number of years monitored ($\chi^2$ for heterogeneity = 7.16. d.f.= 2: 2$P$ = 0.03). rates being highest in workers monitored in 2–4 years (RR = 3.19. 95% CI 1.15–7.54). For workers monitored for plutonium. RRs for all cancers combined increased with the number of years in which workers were monitored ($\chi^2$ for trend = 4.38. 2$P$ = 0.04). For lung cancer. the highest RR was in workers who were monitored for plutonium for 5 or more years (RR = 1.45. 95% CI 1.06–1.96) although neither the heterogeneity nor trend in RRs was statistically significant. There was no statistically significant variation in RRs for all cancers combined. or for cancers of the lung or prostate. with number of years in which workers were monitored for other radionuclides.

There was little suggestion that RRs varied according to age at first monitoring or calendar year of first monitoring in workers monitored for tritium or radionuclides other than tritium or plutonium (Table 5). Among workers monitored for plutonium exposure there was a tendency for RRs for all cancers combined to be higher in earlier calendar periods ($\chi^2$ for trend = 5.91. 2$P$ = 0.02) but none of the calendar period-specific RRs was significantly different from unity.

### Radionuclide monitoring status and cumulative external radiation dose

In order to investigate the potentially modifying effects of external radiation dose on the above findings. RRs were estimated separately for workers receiving less than 10 mSv of external dose and those receiving 10 mSv or more (Table 6). These analyses revealed a statistically significant excess of lung cancer among tritium-monitored workers who had received less than 10 mSv of external

© Cancer Research Campaign 1998

**Table 7** Mortality among radiation workers in three UK workforces (AEA, AWE, Sellafield) in relation to cumulative whole-body dose for all cancers combined and selected cancers according to radionuclide monitoring status. Adjusted for age, sex, calendar period, social class and establishment

| | | Cummulative whole-body dose (mSv)[a] | | | | | | | | z-test for trend[b] Lag period | |
|---|---|---|---|---|---|---|---|---|---|---|---|
| **Cause of death (ICD 8th revision code)** | **Radionuclide monitored for** | **< 10** | **10–** | **20–** | **50–** | **100–** | **200–** | **400+** | **Total deaths** | **0 years** | **10 years** |
| All malignant neoplasms (140–209) | Any | 0.95 (156/163.48) | 0.89 (78/87.82) | 1.07 (162/151.76) | 1.03 (122/118.25) | 1.04 (114/110.09) | 1.14 (108/94.44) | 0.80 (58/72.16) | 798 | − 0.89 | − 0.49 |
| | None | 0.99 (575/582.73) | 0.99 (160/161.26) | 1.00 (191/190.53) | 1.18 (93/78.81) | 0.83 (34/40.88) | 1.05 (29/27.53) | 0.98 (15/15.26) | 1097 | + 0.11 | + 0.22 |
| Bronchus and lung (162) | Any | 1.04 (62/59.47) | 0.97 (30/31.00) | 1.00 (53/53.20) | 1.16 (50/43.16) | 0.95 (41/43.37) | 1.15 (43/37.54) | 0.59 (16/27.25) | 295 | − 2.02 | − 1.50 |
| | None | 1.00 (181/180.11) | 0.78 (42/53.86) | 1.02 (63/62.06) | 1.32 (33/24.99) | 1.34 (17/12.64) | 0.76 (7/9.20) | 0.97 (5/5.14) | 348 | + 0.25 | + 0.29 |
| Pleura (163) | Any | 0.70 (1/1.42) | 0.00 (0/0.97) | 1.16 (2/1.73) | 1.52 (2/1.32) | 2.21 (3/1.36) | 0.81 (1/1.24) | 0.00 (0/0.97) | 9 | − 0.79 | − 0.46 |
| | None | 1.01 (4/3.97) | 1.39 (2/1.43) | 1.31 (2/1.53) | 0.00 (0/0.80) | 0.00 (0/0.59) | 2.45 (1/0.41) | 0.00 (0/0.26) | 9 | − 0.35 | − 0.27 |
| Melanoma and other skin (172–173) | Any | 0.52 (1/1.92) | 0.70 (1/1.44) | 1.16 (2/1.73) | 0.78 (1/1.28) | 2.62 (2/0.76) | 0.00 (0/0.60) | 3.64 (1/0.27) | 8 | + 1.47 | + 1.89* |
| | None | 0.62 (3/4.81) | 1.09 (1/0.92) | 0.00 (0/0.99) | 3.68 (2/0.54) | 2.56 (1/0.39) | 4.10 (1/0.24) | 0.00 (0/0.11) | 8 | + 1.37 | + 2.17* |
| Uterus (180–182) | Any | 1.77 (2/1.13) | 0.00 (0/0.14) | 0.00 (0/1.39) | 3.75 (1/0.27) | 0.00 (0/0.08) | 0.00 (0/0.00) | 0.00 (0/0.00) | 3 | − 0.08 | − 0.94 |
| | None | 1.11 (8/7.21) | 0.00 (0/1.08) | 0.72 (1/1.38) | 2.98 (1/0.34) | 0.00 (0/0.00) | 0.00 (0/0.00) | 0.00 (0/0.00) | 10 | + 0.54 | + 1.02 |
| Prostate (185) | Any | 0.90 (10/11.10) | 0.56 (3/5.31) | 1.09 (12/10.99) | 0.80 (6/7.49) | 1.64 (10/6.08) | 1.57 (8/5.11) | 0.26 (1/3.92) | 50 | − 0.51 | − 1.01 |
| | None | 0.98 (30/30.63) | 0.98 (8/8.19) | 0.89 (11/12.42) | 1.40 (7/5.01) | 0.79 (2/2.52) | 1.54 (3/1.95) | 0.78 (1/1.29) | 62 | + 0.21 | + 0.21 |
| Ill-defined and secondary (195–199) | Any | 1.09 (13/11.96) | 0.81 (6/7.42) | 1.10 (13/11.80) | 0.62 (5/8.04) | 0.57 (4/7.04) | 1.75 (11/6.28) | 1.12 (5/4.46) | 57 | + 1.01 | + 1.12 |
| | None | 0.91 (32/35.26) | 1.18 (9/7.63) | 1.26 (13/10.29) | 0.92 (3/3.25) | 0.55 (1/1.83) | 0.86 (1/1.16) | 1.73 (1/0.58) | 60 | + 0.33 | + 0.48 |
| Multiple myeloma (203) | Any | 1.10 (2/1.82) | 0.00 (0/0.87) | 0.00 (0/1.57) | 2.87 (4/1.39) | 0.99 (1/1.01) | 0.00 (0/1.14) | 1.65 (2/1.21) | 9 | + 0.56 | + 1.09 |
| | None | 0.97 (4/4.12) | 0.82 (1/1.23) | 1.38 (2/1.44) | 0.00 (0/0.67) | 3.24 (1/0.31) | 0.00 (0/0.17) | 0.00 (0/0.06) | 8 | − 0.09 | − 0.66 |
| Leukaemia excluding CLL[c] | Any | 0.53 (1/1.89) | 0.49 (1/2.05) | 1.09 (3/2.76) | 0.89 (2/2.24) | 0.44 (1/2.27) | 2.00 (4/2.00) | 1.66 (3/1.80) | 15 | + 1.67* | + 1.79* |
| | None | 1.09 (20/18.38) | 0.59 (3/5.05) | 0.64 (4/6.24) | 1.65 (4/2.43) | 1.67 (2/1.20) | 0.00 (0/0.53) | 5.64 (1/0.18) | 34 | + 1.43 | + 1.62 |

Results are expressed as ratio of observed to expected deaths [observed and expected numbers of deaths in parantheses; expected deaths are based on all subjects with a radiation record (any or none, as appropriate)]. [a]Numbers shown in body of table are based on a lag period of 0 years. [b]Based on mean exposures in each category weighted according to the distribution of person years at risk in each category: means were 3.38, 14.41, 32.57, 70.80, 141.28, 279.26 and 585.68 mSv for lag = 0 years; 2.83, 14.42, 32.57, 70.80, 141.24, 278.92, 583.18 mSv for lag = 2 years and 1.36, 14.44, 32.54, 70.75, 140.73, 277.10 and 565.45 mSv for lag = 10 years. [c]Lag period of 2 years for leukaemia. Statistical significance, *1 $P < 0.05$.

dose (RR = 1.86, 95% CI 1.01–3.17), but there remained little suggestion of an excess among tritium-monitored workers with doses of 10 mSv or more (RR = 1.05, 95% CI 0.74–1.45). RRs for workers monitored for plutonium exposure appeared relatively unaffected by stratification for level of external radiation dose. The previously noted excess death rate for lung cancer in workers monitored for other radionuclides was present and separately statistically significant for workers with less than 10 mSv and for those with 10 mSv or more of external dose (Table 6). Although the overall excess death rate for prostatic cancer in workers monitored for other radionuclides was evident in both subgroups of workers, neither of the individual RRs was statistically significant.

In our earlier report (Carpenter et al, 1994), we described cancer mortality in relation to cumulative external radiation dose in this cohort of 40 761 radiation workers during the same period of follow-up and found statistically significant positive associations for leukaemia (excluding chronic lymphatic leukaemia, CLL), melanoma and other skin cancers and ill-defined and secondary cancers. Table 7 shows the results of performing similar analyses with additional stratification for radionuclide monitoring status defined as monitoring for any radionuclide vs monitoring for none. It can be seen that death rates from leukaemia excluding CLL and melanoma and other skin cancers increased with external radiation dose in both groups of worker (Table 7). The association for

*© Cancer Research Campaign 1998*

ill-defined and secondary cancers, however, was less evident when stratified by radionuclide-monitoring status. For other specific cancers examined (lung, pleura, uterus, prostate and multiple myeloma) there continued to be little evidence of a trend for either subgroup of worker.

### DISCUSSION

The effects of occupational exposure from internal contamination by radionuclides have been relatively little studied. This contrasts with the large body of results from studies of cancer risks in workers exposed to external radiation. Unlike external radiation, internal doses from radionuclides are likely to be non-uniform across the body and are often extremely difficult to infer. Although monitoring for radionuclide exposure has been carried out routinely in nuclear industry workforces, detailed estimation of doses from internal sources have generally not been made. The main exception in the UK is the Sellafield workforce, for which annual plutonium doses have been assembled for the purpose of a special study and this is to be the subject of a separate report. The analyses described here rely solely on information as to whether or not workers were monitored for exposure to internal contamination by radionuclides. Being monitored for a radionuclide does not necessarily mean that the individual concerned was actually exposed to it. These issues need to be borne in mind when interpreting the results (Atkinson et al, 1994).

Of the few studies that have examined the carcinogenic effects of exposure to radionuclides in nuclear industry workers occupationally exposed to tritium, $^{51}Cr$, $^{59}Fe$, $^{60}Co$ or $^{65}Zn$ were found to be at an increased risk of prostatic cancer, but the separate effects of these individual radionuclides could not be disentangled (Rooney et al, 1993). Many of those workers are included in the present study and so it is not surprising that an excess of prostatic cancer was found here in workers monitored for radionuclides other than tritium or plutonium (Table 2) and that AEA and AWE employees monitored for tritium in 2 or more years had risks two to three times those of radiation workers not monitored for any radionuclide (Table 4). Three deaths from testicular cancer in tritium-monitored male workers constituted a significant excess relative to rates in workers not monitored for any radionuclide, as did three deaths from cancer of the uterus in female workers monitored for radionuclides other than plutonium and tritium (Table 2). These findings are based on a small number of deaths and may have arisen by chance.

The strength of prior evidence for an excess of lung cancer in nuclear industry workers monitored for exposure to radionuclides is not strong for workers in the UK or USA (Wilkinson et al, 1987; Beral et al, 1988; Checkoway et al, 1988; Gilbert et al, 1989; Wing et al, 1991; Fraser et al, 1993). However, workers exposed to plutonium in the radiochemical plant at Mayak, Russia, have a large excess risk of lung cancer (Koshurnikova et al, 1997). Among workers monitored for plutonium exposure in the present study, lung cancer mortality was increased in those who had been monitored for such an exposure for 5 or more years (Table 4). Plutonium-monitored workers also experienced a trend of increasing death rates from all cancers combined in relation to time since first monitoring and duration of monitoring. The trend in relation to time since first monitoring was in part due to a non-significant increase in lung cancer and in part due to a significant increase in other specific cancers, most notably those of the lymphatic and haematopoietic system. Separate analyses currently underway on the Sellafield workforce could provide independent evidence regarding these associations, as well as the opportunity to investigate patterns of cancer risk in relation to the estimated level of plutonium exposure. Death rates from lung cancer in workers monitored for radionuclides other than tritium and plutonium were also 31% higher than those of workers not monitored for any radionuclide (Table 2).

An excess of pleural cancer has been noted previously among radiation workers employed at Sellafield (Douglas et al, 1994). As in the previously reported data relating to the Sellafield workforce, the present study provides no suggestion for a relationship between pleural cancer and external radiation dose (Table 7). All pleural cancer deaths observed were mesotheliomas but, in the absence of data relating to exposure to asbestos, the increased risk of cancer of the pleura in plutonium-monitored workers is difficult to interpret.

As noted above, the lack of dosimetric data for internal exposures has implications for the interpretation of findings reported here. A further consideration is the possible biasing effect that these exposures may have had on previous analyses of mortality in relation to external radiation (Carpenter et al, 1994). In order to examine this issue, analyses of the relation between cancer mortality and external dose were repeated separately for radiation workers monitored for any radionuclide and those monitored for none (Table 7). As before, these analyses continued to provide no suggestion of an association with external dose for all cancers combined whereas, for leukaemia, there was very little evidence that the strength of association differed between these two groups of worker. These findings are broadly similar to those obtained from data for the National Registry for Radiation Workers (which included the majority of workers in the present study), although there was less evidence in the current analyses that the increase in leukaemia mortality with external dose was stronger in workers not monitored for internal contamination (Little et al, 1993).

### CONCLUSIONS

These analyses of cancer mortality in relation to monitoring for radionuclide exposure reported in a large cohort of nuclear industry workers suggest that certain patterns of monitoring for some radionuclides may be associated with higher death rates from cancers of the lung, pleura, prostate and all cancers combined. Some of these findings may be due to chance. Moreover, because of the paucity of related data and lack of information about other possible exposures, such as whether plutonium workers are more likely to be exposed to asbestos, firm conclusions cannot be drawn at this stage. Further investigations of the relationship between radionuclide exposure and cancer in nuclear industry workers are needed.

### ACKNOWLEDGEMENTS

We dedicate this report to the memory of Len Salmon for his invaluable advice and support over the 20 years in which these studies have been conducted. This work was supported by the Medical Research Council by a grant to the Epidemiological Monitoring Unit at the London School of Hygiene and Tropical Medicine and funded by the United Kingdom Atomic Energy Authority, the Atomic Weapons Establishment and British Nuclear Fuels. We thank the staff of these three organizations for their help and advice with this study. We are grateful to the staffs of the NHS Central Registers, the Health Statistics division of the Office of

*© Cancer Research Campaign 1998*

Population Censuses and Surveys and the national insurance records branch of the Department of Social Security at Newcastle. We thank Christine Thorne for secretarial assistance.

### REFERENCES

Atkinson WD, Bull RK, Marshall M, Morgan GR, Newton D and Salmon L (1994). The association between prostate cancer and exposure to $^{65}$Zn in UKAEA employees. *J Radiol Prot* **14**: 109–114

Beral V, Inskip H, Fraser P, Booth M, Coleman D and Rose G (1985) Mortality of employees of the United Kingdom Atomic Energy Authority, 1946–1979. *Br Med J* **291**: 440–447

Beral V, Fraser P, Carpenter L, Booth M and Brown A (1988) Mortality of employees of the Atomic Weapons Establishment, 1951–82. *Br Med J* **297**: 757–770

Cardis E, Gilbert ES, Carpenter L, Howe G, Kato I, Armstrong B, Beral V, Cowper G, Douglas A, Fix J, Fry SA, Kaldor J, Lavé C, Salmon L, Smith PG, Voelz GL, Wiggs LD (1995). Effects of low doses and low dose rates of external ionizing radiation: cancer mortality among nuclear industry workers in three countries. *Radiat Res* **142**: 117–132

Carpenter L, Higgins C, Douglas A, Fraser P, Beral V and Smith P (1994) Combined analysis of mortality in three United Kingdom nuclear industry workforces, 1946–1988. *Radiat Res* **138**: 224–238

Checkoway H, Pearce N, Crawford-Brown DJ and Cragle D (1988) Radiation doses and cause-specific mortality among workers at a nuclear materials fabrication plant. *Am J Epidemiol* **127**: 255–266

Douglas AJ, Omar RZ and Smith PG (1994) Cancer mortality and morbidity among workers at the Sellafield plant of British Nuclear Fuels. *Br J Cancer* **70**: 1232–1243

Fraser P, Booth M, Beral V, Inskip H, Firsht S and Speak S (1985) Collection and validation of data in the United Kingdom Atomic Energy Authority mortality study. *Br Med J* **291**: 435–439

Fraser P, Carpenter L, Maconochie N, Higgins C, Booth M and Beral V (1993) Cancer mortality and morbidity in employees of the United Kingdom Atomic Energy Authority, 1946–86. *Br J Cancer* **67**: 615–624

Gilbert ES, Petersen GR and Buchanan JA (1989) Mortality of workers at the Hanford site: 1945–1981. *Health Phys* **56**: 11–25

Gilbert ES, Cragle DL and Wiggs LD (1993) Updated analyses of combined mortality data for workers at the Hanford Site, Oak Ridge National Laboratory and Rocky Flats Weapons Plant. *Radiat Res* **136**: 408–421

Inskip H, Beral V, Fraser P, Booth M, Coleman D and Brown A (1987) Further assessment of the effects of occupational radiation exposure in the United Kingdom Atomic Energy Authority mortality study. *Br J Indust Med* **44**: 149–160

Koshurnikova NA, Shilnikova NA, Okatenko PV, Kreslov VV, Bolotnikova MG, Romanov SA and Sokolikov ME (1997). The risk of cancer among nuclear workers at the 'Mayak' production association: preliminary results of an epidemiological study. In: *Implications of New Data on Radiation Cancer Risk.* Proceedings of the 32nd annual meeting of the National Council in Radiation Protection and Measurements, 3–4 April, 1996, Boice JD (ed.), pp. 113–122

Kendall GM, Muirhead CR, MacGibbon BH, O'Hagan JA, Conquest AJ, Goodill AA, Butland BK, Fell TB, Jackson DA, Webb MA, Haylock RGE, Thomas JM and Silk TJ (1992) Mortality and occupational exposure to radiation: first analysis of the National Registry for Radiation Workers. *Br Med J* **304**: 220–225

Little MP, Kendall GM, Muirhead CR, MacGibbon BH, Haylock RGE, Thomas JM and Goodill AA (1993) Further analysis, incorporating assessment of the robustness of risks of cancer mortality in the National Registry for Radiation Workers. *J Radiol Prot* **13**: 95–108

Lubin JH, Boice JD, Edling C, Hornung RW, Howe GR, Kunz E, Kusiak RA, Morrison HI, Radford EP, Samet JM, Tirmarche M, Woodward A, Yao SX, Pierce DA (1995) Lung cancer in radon-exposed miners and estimation of risk from indoor exposure. *J Natl Cancer Inst* **87**: 817–827

National Research Council (1988) Health risks of radon and other internally deposited alpha-emitters. BEIR IV. National Academy Press, Washington, DC

Office of Population Censuses and Surveys (1970) *Classification of Occupations 1970.* HMSO: London.

Preston DL, Lubin JH, Pierce DA and McConney ME (1993) *Epicure User's Guide.* Hirosoft International Corporation: Seattle

Rooney C, Beral V, Maconochie N, Fraser P and Davies G (1993) Case–control study of prostatic cancer in the United Kingdom Atomic Energy Authority employees. *Br Med J* **307**: 1391–1397

Smith PG and Douglas AJ (1986) Mortality of workers at the Sellafield plant of British Nuclear Fuels. *Br Med J* **293**: 845–854

UNSCEAR (1994) *Sources and Effects of Ionizing Radiation: UNSCEAR 1994 report to the General Assembly with Scientific Annexes.* United Nations: New York

Wilkinson GS, Tietjen GL, Wiggs LD, Galke WA, Acquavella JF, Reynes M, Voelz GL and Waxweiller RJ (1987) Mortality among plutonium and other radiation workers at a plutonium weapons facility. *Am J Epidemiol* **125**: 231–250

Wing S, Shy CM, Wood JL, Wolf S, Cragle DL and Frome EL (1991) Mortality among workers at Oak Ridge National Laboratory: evidence of radiation effects in follow-up through 1984. *J Am Med Assoc* **265**: 1397–1402

World Health Organization (1967) *International Classification of Disease, Injuries and Causes of Death*, 8th revision, 1965. WHO: Geneva

World Health Organization (1977) *International Classification of Diseases, Injuries and Causes of Death*, 9th Revision, 1975. WHO: Geneva

 © Cancer Research Campaign 1998